# An examination of the social determinants of health as factors related to health, healing and prevention of foetal alcohol spectrum disorder in a northern context – the brightening our home fires project, Northwest Territories, Canada

**DOI:** 10.3402/ijch.v72i0.21140

**Published:** 2013-08-05

**Authors:** Dorothy Badry, Aileen Wight Felske

**Affiliations:** 1 Faculty of Social Work, University of Calgary, Calgary, AB, Canada; 2 Canada FASD Research Network Action Team on Women's Health Member, Yellowknife, NT, Canada; 3 Mount Royal University, Calgary, AB, Canada

**Keywords:** foetal alcohol spectrum disorder, prevention, social determinants of health, Northwest Territories, women's health, qualitative research, alcohol, northern health

## Abstract

**Objective:**

The Brightening Our Home Fires (BOHF) project was conceptualized as an exploratory project to examine the issue of the prevention of foetal alcohol spectrum disorder (FASD) from a women's health perspective in the Northwest Territories (NT). While dominant discourse suggests that FASD is preventable by abstention from alcohol during pregnancy, a broader perspective would indicate that alcohol and pregnancy is a far more complex issue, that is, bound in location, economics, social and cultural views of health. This project was prevention focused and a social determinant of health (SDH) perspective informed this research.

**Methods:**

The BOHF project was a qualitative research project using a participatory action research framework to examine women's health and healing in the north. The methodology utilized was Photovoice. Women were provided training in digital photography and given cameras to use and keep. The primary research question utilized was: *What does health and healing look like for you in your community*? Women described their photos, individually or in groups around this central topic. This research was FASD informed, and women participants were aware this was an FASD prevention funded project whose approach focused on a broader context of health and lived experience.

**Results:**

This project drew 30 participants from: Yellowknife, Lutsel ‘ke, Behchokö and Ulukhaktok. These four different communities across the NT represented Dene and Inuit culture. The qualitative data analysis offered themes of importance to women's health in the north including: land and tradition; housing; poverty; food; family; health, mental health and trauma, and travel. Photovoice provides a non-threatening way to engage in dialogue on complex health and social issues.

The purpose of the Brightening Our Home Fires (BOHF) research was to engage with women in their home communities in northern Canada in relation to their beliefs and attitudes towards healthy living. It is recognized that individual and community experiences amongst women are critical to understanding new social perspectives of health determinants in northern Canada. While this research was positioned as an exploratory fetal alcohol spectrum disorder (FASD) prevention project, it was clear that the issue of FASD can be constructed as a product of many other factors that lead to women using alcohol to self-medicate against social and health problems. FASD has been broadly defined as a disabling condition that has multiple presentations including a primary lifelong organic brain injury caused by prenatal alcohol exposure. Alcohol consumption is commonly known as a means by which women buffer their own pain and trauma (1) and is a public health issue. Photovoice, a qualitative methodology, offered a way to gently engage with women on issues that were important within their lives and specifically in relation to health (Fig. 1).

**Fig. 1:**
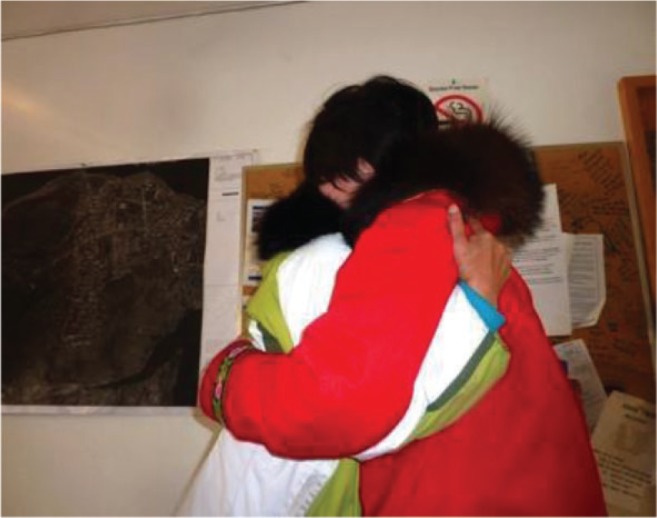
A reunion at the Kugluktuk Airport with a project participant and a relative. Photo credit: Dorothy Badry.

## Objectives of the research

- To identify voices of women in their home community through Photovoice work.
- To understand factors that northern women see as contributing to their health and healing in their own community.
- To understand what is unique to each community specifically for women in understanding health and healing and to identify factors, that may be protective and can potentially, contribute to FASD prevention grounded in a women's health framework.

The BOHF project represents the voices of women in the NT and their health concerns with a background focus on FASD prevention. The decision to engage in this research was driven by a need identified in the Northwest Territories (NT) from members belonging to the Canada FASD Research Network Action Team (NAT) on Women's Social Determinants of Health. This project received ethics approval from the Conjoint Faculties Research Ethics Board of the University of Calgary and the Aurora Research Institute in the NT. The effectiveness of available FASD prevention information, generally perceived as stigmatizing by pregnant women was not viewed as effective, while the need to explore prevention existed.

In terms of context, going into a remote community and simply opening discussions about FASD prevention was not considered to be an approach that would be successful by NAT members located in northern communities. With this in mind, the primary research question that emerged as guiding this project was: *What does health and healing look like for you in your community?* This question encouraged participation as it was considered relevant and non-threatening, and did not directly ask about alcohol use and pregnancy. However, signing informed consents and identifying BOHF as an FASD prevention project provided a window to at least raise the topic and some discussion amongst participants and research team members.

## A brief review of relevant literature

As this project was housed in a northern context, literature related to the SDH and implications for women's health were reviewed. The BOHF research was reflective of a gendered position in terms of understanding women's health as influenced by context, place and experiences of northern women. Benoit and Shumka (2) consider the roles of sex and gender in relation to the health of girls and women in light of the power these constructs hold in determining health outcomes. Of particular relevance to the BOHF project is the model proposed by Benoit and Shumka (2) that considers “sex and gender on equal footing with other fundamental determinants, including race, ethnicity … geographical location and age” (p. 10). In remote communities, girl's and women's health, practices and beliefs are intricately interwoven across generations and culture. Health disadvantages exist in the north with geography acting as a critical factor in opportunities to accessing health supports.

## Social determinants of health

Canada views health, as not just a state, but also a “resource for everyday life” (3, p. 9). Population research indicates that the key factors which influence health are: income and social status; social support networks; education; employment/ working conditions; social environments; physical environments; personal health practices and coping skills; healthy child development; biology and genetic endowment; health services; gender; and culture. Health of populations can be examined by age, gender, ethnicity or region. Such a focus reveals that health inequities are experienced by Canadians in certain areas of Canada and by certain groups including the north. As a concept, SDH have recently been readopted (4) with both a preventative (problem) and treatment focus.

## Social determinants of health in the north

An early case study by Hildes, Whaley, Whaley, Irving (5), a team of medical researchers on behalf of the Arctic Health Research Centres, studied the physiological response to cold in Old Crow, Yukon (Vuntut Guichen), as well as the state of health and well-being of individual community members. An overwhelming number of people in this small northern community were in good health and experiencing positive social well-being. Fifty years later, after the introduction of residential schooling and alcohol to northern life, a different picture emerges. Parlee, O'Neil and Lutsel ‘Ke Dene First Nation (6) examined the meaning of health within the context of Canada's first diamond mine located in the territory of this community. This study examined health through questions such as; “What is a healthy community? What is it about the Dene way of life, that is, so important?” (p. 118). A key finding suggested that “many community members perceive that people were healthier when they were living on the land” (6, p. 116). Loppie, Wien (7) documented the health inequalities of Aboriginal people's lives in Canada and proposed a model of organizing the SDH, as distal (e.g. historic, political, social and economic contexts); intermediate (e.g. community infrastructure, resources, systems and capacities); and proximal (e.g. health behaviors, physical and social environment). The complexity of SDH attracts researchers in the areas of social and community engagement, as it potentially offers a new avenue of solutions.

Cameron (8) identified emerging health trends in Inuit communities including rising rates of diabetes and nutritional concerns as less traditional diets are followed. Other concerns include increasing sexually transmitted infections, youth suicide and issues related to intergenerational trauma. Cameron offers a contemporary review of the SDH and indicates that health is largely linked to socio-economic factors including: acculturation, productivity, income distribution, housing, education, food security, healthcare services, social safety nets, the quality of early life, addictions and the environment. What is the bridge that links FASD as part of the picture of health?

## An FASD lens

FASD prevention is best addressed in First Nations and Inuit communities from a cultural, historic, political and social context and often takes different forms from mainstream approaches (9) for reasons of geography and access to health-related resources. Poole (10) indicated that women's alcohol use is not just about alcohol. It is also about stress, context, isolation, general health, age, genetics, resilience, cultural discrimination, exposure to violence, abuse, access to prenatal care, grief and loss, social policy and poverty. Approaching FASD from a cultural lens, that is, respectful of history while moving a women's health agenda forward, positions FASD as a health problem requiring supports for at-risk families with a goal of child and family health wherever families reside in Canada.

## Methodology

**Fig. 2:**
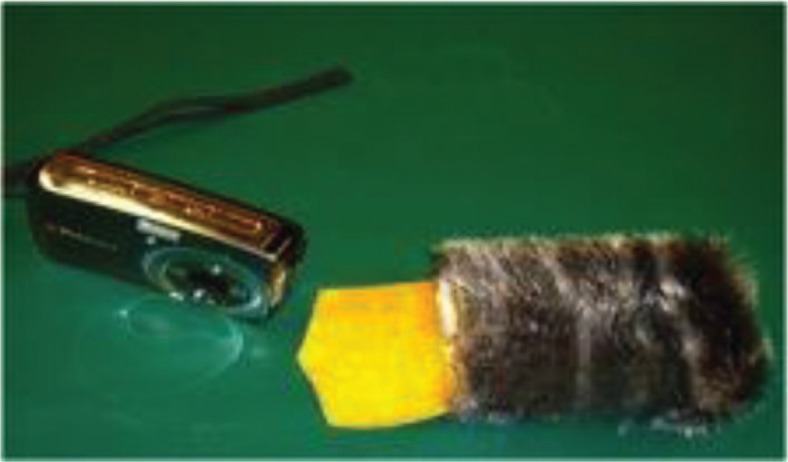
A camera case made overnight by a project participant in Ulukhaktok. Photo credit: Dorothy Badry.

Photovoice is a participatory action research approach to working in communities on health and social issues and position participants as co-researchers of the study. Digital cameras were provided to women participants and team members offered a community workshop on using the cameras and taking pictures. Once photos were taken, a meeting took place and for most women, their photos were discussed and captions developed individually rather than in a group setting. In a community a translator was needed. Atlas Ti, a qualitative software programme was utilized to consolidate, review and code text to support developing a portrait and themes in response to the question: *What does health and healing look like for you in your community?* (Fig. 2)

## Results

Through the BOHF research and based on the findings from a depth analysis of key themes, text and images, the SDH were re-examined with a perspective contextually grounded in northern Canada. The SDH provided an anchor and framework to consider and examine the photovoice findings of this project on health, healing and FASD prevention for women in northern Canada.

### Key themes emerging from the brightening our home fires project

**Fig. 3:**
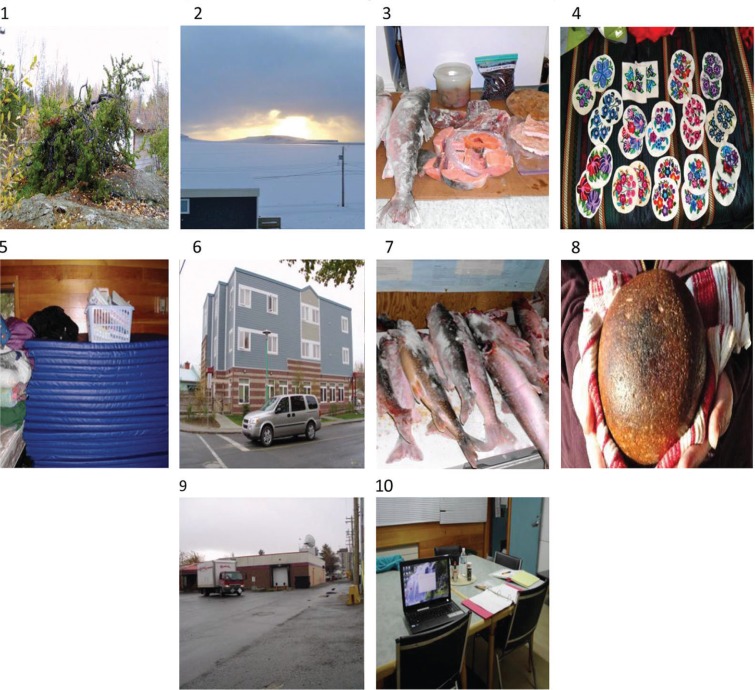
Brightening our home fires image panorama.

Key themes that emerged from the project in relation to an SDH framework included: land and tradition; housing, poverty, food, family, health, mental health and trauma, and travel. An additional discussion with a participant provided insight into how travel in the north potentially affects health needs. Within the limitations of this paper, we briefly present captions and images from these findings through the voices of women participants. Women experiencing homelessness in Yellowknife shared images that were quite divergent from women living in a community. This panorama of photographs serves to illustrate how images portray places and different experiences (Fig. 3).

In relation to the key themes, a few illustrative captions from the panorama of photographs are shared below in the order that the images are presented.

### Theme: land and tradition

Image 1: The tree. *The tree clears the air and it's healthy. It's part of nature, part of the world. We need the tree. It makes me happy because it looks nice there. It makes it nice for the city. It makes me feel sad for the tree being in the cold weather. It is challenging to be in the cold. I've been out in the cold and it's cold, very cold*.

Images 2 & 3: Our land. *Looking out my front window – our country food – Arctic char, berries, a round bannock type bread and soups*.

Image 4: My work – my handicrafts. *Part of my therapy is through my handicrafts, which are very relaxing in creating the colors and the choice of colors – the colors I choose reflect my healing and how far I have come. I have a need to see the mistakes I make sometimes in my own life and reflect on how beautiful life on my own life can be through my creations. Sometimes it 's so easy to go back and see the mistakes in life and as I do my creations through my crafts it brings me right back up. I do not stay too long in my own negative world I used to be in, because it was back in my lifetime, the time of darkness and pain. As I do my handicrafts it helps me to relax and take pride in my work and be real in my own healing* journey …

### Theme: housing/poverty

Image 5: Plastic bin and mattresses at the Centre for Northern Families in Yellowknife. *I'd like to find a better place. There is no room – too close together, have to fold mats, women shower and go early before fights start, clothes, more luggage, have to wipe clean mats – some women leave and don't help. Clothes; dirty and clean – all mixed up, no room. I used to have a place*.

### Theme: food

Image 6: Inuit Boarding Home (Larga House) near downtown Yellowknife. *This clinic is for pregnant ladies and other patients. They have Inuuk food; fish, char caribou, musk ox, and polar bear. When you are from out of town you are allowed to live there when you are a patient. It's important for Inuit to have that clinic. I was in the old clinic for medical help. I got bedding, a room, I had food there. That helped*.

Image 7: Arctic char frozen in a porch at the Kayuktuk Centre, Ulukhaktok, NT. *Community Spirit of Sharing. This fresh catch of Arctic Char was harvested by two local fisherman hired by the local hunters & trappers organization. The fish is distributed amongst the local community members. Sharing of country or native foods is part of our Inuvialuit culture, especially to elders and single women with children*.

### Theme: health, mental health and trauma

Image 8: Medicine Rock. *Medicine rock. Keep it heated on the stove. Many uses. Warmth in a pack when out on the land; calming, relaxing, pain relief. It is precious to me. Medicine Rock – warming my hands*.

Image 9: Back alley. *We go main street – meet friends outside the bar who will give me a shot of drinks. Sometimes [there's] nothing to do – back to the same old place. People are jealous and put me down. Say things like “she's too good” or making other friends not accepted. Sometimes the women fight and call each other down, even sober*.

Image 10: Table and computer at the Centre for Northern Families, Yellowknife, NT. *This is the first thing I'm starting on – just trying to learn one day at a time – working on those things like learning computers instead of being out there and enjoying myself (being out there and having a party and not trying to think twice about going the right way in life). This life – doing simple things to keep my mind away from other things I have done in my life. If I just stay forward hopefully there is something out there that will keep my mind off drinking and find a simple thing to keep me doing other things I want to do in my life. May the good spirit always be with us*.

### Theme: travel

This quote emerged from a discussion with a woman participant who also acted as a translator in a remote community and highlighted the need to consider travel as a social determinant of health in a northern context.

*Many times people's health deteriorates as they wait for appointments. Even for alcohol treatment issues that are acute, the need must be expressed, you ask for help and wait for the next approval from the headquarters of the regional office, make an appointment, find out if beds are available and have to wait for an available bed … [and] the process takes a long period of time. Treatment for youth at risk is also a long waiting time and experiences of chaos and abuse occurring within families and overwhelming family units while trying to take care of the person, while trying to maintain some routine in the family. The worker in the community comes up against unseen walls of red tape … in trying to find the space and the right treatment centre– not just availability but who will pay*.

This quote illustrates the need to consider the role of travel as fundamental in relation to determining health. We believe travel should be considered as a new social determinant of health in northern, remote and isolated communities in Canada.

## Conclusions

The BOHF research project was initiated by members of the Canada FASD Research Network Action Team on Women's Social Determinants of Health located in the NT. It offered additional resources related to consultation, professional research expertise drawn from the NAT membership and provided on-going support for the project. While the BOHF project was exploratory in relation to FASD prevention, it was evident that a focus on women's health as contextualized in place and environment such as communities in northern Canada is crucial to develop awareness and programmes. The construct of “place-based research” put forward by Parlee et al. (6, p. 129) is important as “place” has clearly distinct meanings between disenfranchised, homeless women in a more urban setting in Yellowknife than other women participants living in their home communities.

FASD prevention work must be community specific and wrapped in a healthy families envelope from a policy perspective. Focused prevention work must begin earlier than first contacts with the medical system regarding pregnancy and must be linked with the dominant views of health in particular communities, mindful of tradition, culture and the high value placed on connections to children, family and community. Women who are disenfranchised from their communities through homelessness face many challenges and should have access to similar resources such as local food and traditional activities. Experiences on the land and related to the land were identified as particularly important for women in the north. A need exists to highlight the importance of access to local foods (high-protein meat) during pregnancy, and an avoidance of “southern risks” such as alcohol and processed foods high in glucose and carbohydrates could be highlighted as a prevention activity. A focus on overall general health for women supports FASD prevention. Programmes and policies could be expanded that support low stress, land and cultural focused activities that are seen as important during a pregnancy.

The images and text generated in the BOHF project offer a glimpse into social concerns of the north including homelessness, poverty, health-related services, historical trauma, geographical barriers, housing and challenges in social safety nets in areas such as food security. The other construct emerging from this research is a portrait of connectedness to the land, traditional activities, culture, country foods, as well as strengths and resiliency grounded in community and place. We believe that photovoice offers a convincing research model to assist and support the involvement of northern women in Canada in important health discussions respectful of identity, place and culture. A pathway to FASD prevention and awareness programmes in northern, remote and isolated communities in Canada must be location-specific and designed and driven by community members themselves. In its delivery, FASD prevention programmes in the north are best served by local supports through constructing an agenda or programme of FASD awareness and prevention grounded in culture and a northern identity.
